# Appraisal Bias and Emotion Dispositions Are Risk Factors for Depression and Generalized Anxiety: Empirical Evidence

**DOI:** 10.3389/fpsyg.2022.857419

**Published:** 2022-07-04

**Authors:** Klaus R. Scherer, Marco Costa, Pio Ricci-Bitti, Valérie-Anne Ryser

**Affiliations:** ^1^Department of Psychology and Swiss Center for Affective Sciences, University of Geneva, Geneva, Switzerland; ^2^Department of Psychology, University of Bologna, Bologna, Italy; ^3^FORS – The Swiss Centre of Expertise in the Social Sciences, University of Lausanne, Lausanne, Switzerland

**Keywords:** risk for depression, appraisal bias, emotion disposition, coping potential, survey data

## Abstract

Appraisal theory of emotion predicts that appraisal biases may generate stable emotion dispositions, which can ultimately lead to affective disorders. One example is the habitual underestimation of one’s potential to cope with adverse events, which favors frequent experiences of sadness and worry and therefore increases the risk for development of depression and generalized anxiety disorders. To examine the relationships between these variables as potential risk factors, in Study 1, we used appraisal and emotion questions in the Swiss Household Panel (SHP), a nationwide representative sample, and analyzed data for *N* = 4,859 participants in one annual survey wave (Wave 14, SHP 2012) *via* theory-based hierarchical regressions. Path analysis of the nomological network linking frequent experiences of depression and anxiety to the emotion dispositions of sadness and worry, and measures of perceived coping potential (appraisal bias) supports the theoretical predictions and further identifies the effects of important background variables such as personality, motivation, and life events. Discriminant analysis shows that these predictors allow correct classification of close to 70% of the participants with elevated risk. In Study 2, we used established validated instruments to assess the risk for depression and anxiety disorders, as well as a recently validated scenario method to assess appraisal bias and emotion disposition in a survey with *N* = 152 students. The results correspond to the theoretical predictions and largely confirm the findings with the household survey. The results of both studies demonstrate the utility of using current emotion theory to provide new vistas for research on risk factors for affective disorders and to inform the development of appropriate interventions to reduce the level of risk.

## Appraisal Bias as a Risk Factor for Depression and Generalized Anxiety: Empirical Evidence

Depression and generalized anxiety disorders (GAD) are the two most frequent types of mental illness worldwide, with a steadily rising incidence (especially among younger adults). It is thus not surprising that there is an enormous literature on the risk factors involved. A recent umbrella review of evidence from 134 meta-analyses spanning 1,283 studies revealed convincing evidence for six risk factors: widowhood, physical abuse during childhood, obesity, having 4–5 metabolic risk factors, sexual dysfunction, and job strain (Köhler et al., 2018). In addition to stressful life events and interpersonal problems (see also, Hettema et al., 2006; Hammen, 2018), psychologists have underlined the important role of personality, in particular neuroticism (Kotov et al., 2010; Sutton et al., 2011; Vittengl, 2017), and negative versus positive trait affect (Watson and Clark, 1984; Watson et al., 1988; Clark and Watson, 1991; Dejonckheere et al., 2018). A central research domain addresses individual differences in the form of various cognitive vulnerabilities (Teasdale, 1988; Murphy et al., 1999; Mathews and MacLeod, 2005; Everaert et al., 2012). Among the major theoretical models proposed in this domain are the following: the helplessness model (Seligman, 1975; Abramson et al., 1978), the hopelessness theory (Abramson et al., 1989), the response styles theory (Nolen-Hoeksema, 1991), self-serving attributional bias (Sweeney et al., 1986; Greenberg et al., 1992; Haidt and Rodin, 1999; Sanjuán and Magallares, 2009), and self-efficacy beliefs (Bandura et al., 1999; Muris, 2002). Recent reviews of empirical studies in this domain show the relative prevalence of pessimistic inferential style, dysfunctional attitudes, and rumination for depression and of anxiety sensitivity, intolerance of uncertainty, and fear of negative evaluation for anxiety (Hong and Cheung, 2015) as well as high neuroticism, low self-esteem, and negative repetitive thinking (Struijs et al., 2021).

Most of these models for *emotion disorders* have been developed and empirically studied in the domains of psychiatry and personality or clinical psychology, with little input from *emotion psychology*. Thus, one important issue that remains to be explored is the *mediating role of specific “normal” emotions* such as sadness and worry in the underlying mechanism. As part of the debate about the criteria in the new guidelines of the fifth edition of the *Diagnostic and Statistical Manual of Mental Disorders* (American Psychiatric Association, DSM-5 Task Force, 2013), it has been argued that emotion theory and research can provide major input into a better understanding of the *transition from normal to abnormal emotions* that potentially result in severe emotional disorders (see the special issue of *Emotion Review*, Scherer and Mehu, 2015; Table of Contents listed in Appendix 4 of the online supplemental materials). After all, it seems reasonable to assume that in order to study disorders and dysfunctions of psychosomatic dysfunctions, it would be helpful to understand functioning under “normal” conditions, that is, in everyday life, in a random sample population (e.g., a household panel) so that the factors that can be considered risk factors for abnormality may be examined.

Appraisal theories of emotion (Smith and Lazarus, 1993; Roseman, 2001; Scherer, 2001, 2009; Scherer et al., 2001; Ellsworth and Scherer, 2003; Moors et al., 2013) can provide a major contribution to such efforts. Appraisal theorists assume that emotions are elicited and differentiated by the subjective evaluation, based on a number of appraisal criteria, of the personal significance of events and one’s ability to deal with the consequences. Apart from the central issue of whether the consequences of an event are good or bad for reaching one’s goal, two major appraisal criteria are *control* (Can human agency control the consequences?) and *power* (Do I have sufficient power to change the consequences?). If the results of these appraisals are realistic, the resulting emotion is likely to be functional (e.g., with respect to producing appropriate action tendencies). If this is not the case, the emotion is likely to be dysfunctional and maladaptive. If there is a tendency or bias toward repeatedly making unrealistic appraisals, for example, by over- or underestimating one’s coping potential, a more enduring emotional disorder may develop (see also Kuppens and Tong, 2010). Thus, Scherer (1987, p. 77) proposed the following hypotheses:

Mania          overestimation of control and power

Anxiety disorders      concern about adequacy of power, but feeling that control is possible

Hopelessness       underestimation of control

Helplessness      underestimation of power

On the basis of this early proposal, Scherer and his collaborators have suggested that cognitive *appraisal biases* may be responsible for the development of dysfunctional *emotion dispositions*, that is, the tendency to experience certain emotions more frequently than warranted, constituting major risk factors for emotional disorders (Scherer, 1989, 2015; Scherer and Brosch, 2009; see also Roseman and Kaiser, 2001). It should be noted that the appraisal bias concept differs from the more general term “cognitive bias” in that it focuses specifically on very concrete appraisal steps such as valence, coping/control, causation, etc. in the judgment process. In contrast, general cognitive bias includes attention biases, working memory difficulties, and long-term memory biases (Everaert et al., 2020). Similarly, the widely studied domain of cognitive vulnerabilities covers many different types of maladaptive beliefs and attitudes, processing and thinking styles, as well as self-image (Hong and Cheung, 2015; Struijs et al., 2021). Clearly, all of these may be affected by appraisal bias or, in turn, affect appraisal bias. Thus, low self-esteem may be due to low control/coping beliefs or be partly responsible for low coping/control appraisal bias. Sander et al. (2005, p. 321–322) have discussed some of these bidirectional effects between appraisal and other cognitive functions such as stored schemata, representations in memory and self-concept, and expectations. However, the etiology of appraisal biases needs to be clarified by further research in this domain. Here we assume the existence of appraisal biases as a dispositional concept, which is expected to have a direct impact on the nature of the ensuing emotion and on emotion dispositions as well as emotion disorders.

Appraisal researchers have developed a theoretical framework that proposes concrete predictions on the type of appraisal bias likely to produce a particular form of emotional disorder (for further details, see Scherer and Brosch, 2009, and Supplementary Table S1). Specifically, they hypothesize that an appraisal bias of consistently underestimating one’s ability to control and cope with the consequences of adverse events across many different situations increases the frequency of experiences of worry and sadness and thus facilitates the development of depression and GAD.

These suggestions, developed from the theoretical framework of appraisal theory of emotion, are highly compatible with the large number of approaches, cited earlier, that highlight cognitive vulnerability as a major risk factor for affective disorders. Mehu and Scherer (2015) provide a brief overview and discussion of the relationship of these theories to the appraisal bias model. They argue that the main difference between the appraisal bias model and other models lies in the temporal position—within the flow of events leading from life challenges to depressive symptoms—of the respective cognitive processes. Whereas earlier models emphasize the role of individual differences in information processing *after* the negative emotional experience is initiated, the appraisal model argues that consistent appraisal biases in the way that events are processed in the first place increase the tendency to experience episodes of sadness and despair and thus increase the risk of developing clinically relevant depression. In other words, there is a slow buildup of an ever-increasing tendency toward “sadness orientation,” progressing from normal to pathological. Thus, the major difference is that the predictions concerning the risk for depression and anxiety mood disorders based on an appraisal bias model assume a *mediating role for stable emotion dispositions*. The assumption is that individuals for whom a low coping potential appraisal bias (underestimating both control and power potential) has created a stable emotion disposition to experience sadness and/or worry more frequently and more strongly will be at greater risk of suffering clinically relevant depression or anxiety disorders.

The first step in examining this mediation hypothesis is to establish the existence of emotion dispositions that can be reasonably attributed to appraisal bias. The first evidence for the existence of emotion dispositions was obtained in a semi-representative survey of emotion experiences in the population living in Switzerland (*N* = 1,242; Scherer et al., 2004). It corroborated the notion that an emotion disposition may induce a tendency to experience certain emotions more frequently. To assess emotion dispositions, respondents rated the relative frequency with which they had experienced each of 14 emotions in the past months. Respondents then reported an event that had elicited an emotion on the previous day. Those who frequently experienced a particular emotion in their daily life were three times more likely (odds ratio) to have experienced the corresponding emotion yesterday. Furthermore, respondents scoring high on the eating disorder, depression, and anxiety disorder scales in a self-report version of the Primary Care Evaluation of Mental Disorders questionnaire (Spitzer et al., 1999) also showed increased odds ratios of having experienced anxiety yesterday. The strength of the emotion disposition effect is surprising, as a major emotion experienced “yesterday” can generally be accounted for by a large number of different factors (see Epstein, 1979). Even though these results are not surprising, they underline the need to better understand the nature and origin of *emotion dispositions* as separate from *trait emotionality* (see also Tangney, 1990; Reisenzein and Weber, 2009).

More recently, Scherer (2021) reported two large-scale studies on emotion dispositions and appraisal bias that used a scenario imagination assessment method. Study 1, with 3,052 professionals in assessment sessions, confirmed the existence of stable emotion dispositions and showed important correlations with several personality traits. Concerning the central role of appraisal bias, in Study 2, representatively selected participants in a US web panel (*N* = 190) were asked to indicate how they would most likely *appraise the events* in nine realistic scenarios, in addition to the *emotions they would most likely experience* in the respective situations. The scenario measurement allows a more fine-grained determination of emotion dispositions and appraisals by focusing on the frequent reoccurrence of certain appraisal and emotion responses across different scenarios, rather than on simple self-report of the estimated frequency with which one experiences certain emotions or makes certain types of evaluations. The results confirmed the theoretical prediction that specific appraisal biases do in fact account for a large percentage of the variance in the expected emotion responses. In particular, the reporting of worry and sadness as emotions anticipated to be felt by the individual in several of the realistic scenarios generally coincided with a conjectured appraisal of low coping potential (see Supplementary Table S1).

In this article, we are extending this approach to the area of affective disorders by examining whether appraisal bias and related emotion dispositions should be considered risk factors for developing depression or GAD. From the theoretical framework outlined earlier, we specifically predict that an appraisal bias resulting in habitual underestimation of one’s capacity to cope with negative events increases the tendency to frequently experience sadness or worry. In turn, these emotions generated by inappropriate appraisal may give rise to a clinically relevant increase in feelings typical of depression and anxiety. We further predict that a number of background variables (such as biographical factors, personality, motivation, and negative life events in critical phases) can affect both emotion dispositions and (directly or mediated *via* these dispositions) the tendency to frequently experience a state of depression or anxiety.

For both ethical and practical reasons, these hypotheses cannot be tested in experimental studies by using systematic manipulations. Realistically, we are limited to a self-report approach. Most earlier work used self-report scales to study clinically relevant groups. Although this approach produces important insights, it needs to be complemented by studies on large, randomized, representative samples of the general population. Such studies are even more indicated because it is important to understand the mechanism involved in a “normal” sample (i.e., a random sample from a general population) before the first indicators are detected.

In this article, we present first empirical evidence for the hypothesis that appraisal bias and associated emotion dispositions of sadness and worry can be potential risk factors for the development of depression and general anxiety obtained in two successive studies. In Study 1, we used data from a single survey wave of an official Swiss nationwide household panel survey (based on telephone interviews) with a large representative group of participants. To obtain the first cross-sectional evidence for the theoretical predictions outlined earlier, we based our analyses on short forms of established psychometric instruments. The inherent limitations of scales with a limited number of items is compensated by the possibility of obtaining data from a very large representative sample of the population. In order to test the replicability of the results with standard multi-item scales, we ran a second study, Study 2, where we measured the major variables *via* established psychometric instruments and a recently validated scenario-based assessment instrument, using a sample of young adults.

Obviously, these cross-sectional studies do not allow to test the causal mechanism suggested by the theoretical assumptions. To firmly establish proof of causality, experimental manipulation or intervention is required. Longitudinal designs help but cannot provide absolute certainty, either. In many cases, as in the current one, experimental manipulation or intervention is impossible for ethical reasons and low feasibility. The latter is also a major issue for longitudinal designs. Most longitudinal studies in the domain of emotional disorders concern therapeutic interventions. The proposed causal path from appraisal bias to emotion dispositions to risk for emotion disorders is most likely a very complex process, probably involving recursive influences, that extends over many years. At present, we know very little about the factors involved, their interaction, and the temporal unfolding. This is why any longitudinal design needs input about the important factors to measure and the time frames to be chosen. Given that there is insufficient information on these factors so far, input from cross-sectional data that establishes solid, replicated statistical associations can help to identify effect sizes for potential parameters and their interdependencies. This is how we understand the contribution of the research reported here.

## Study 1

**Figure 1:**
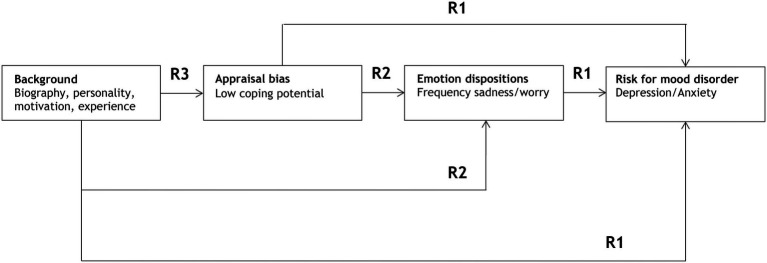
Conceptual representation of the causal path model and sequence of hierarchical regression analyses (R) determining direct effects on risk for mood disorder (R1), emotion disposition (R2), and appraisal bias (R3). R1, R2, R3 refer to three consecutive regression analyses in a theoretically predicted order.

The multidisciplinary longitudinal Swiss Household Panel (SHP) survey (Tillmann et al., 2016; SHP Group, 2022) is ideal for our purposes, as the regular interview schedule already contains questions for many psychological constructs involved in the theoretical framework, such as questions on moods, beliefs, and suffering from life events, as well as motivation and personality traits. Notably, at an early stage of the development of the survey, we had the chance to include four items that measure the frequency of experiencing sadness, worry, anger, and joy (with standard self-report questions, as a scenario measurement cannot be used in lengthy multidisciplinary and generalist telephone interviews). The analysis of the survey data was structured in such a way that different classes of variables could be differentiated with respect to their position in a causal path model that combined several classes of variables with differential proximality to the major dependent variable, risk for mood disorder (see Figure 1). These classes are, in descending order of proximality, as follows: (1) mental predispositions (emotion dispositions, severity of suffering), (2) cognitive appraisal bias (coping potential), and (3) background factors (socio-demographic, personality, motivation, and past experience). We decided to include severity of suffering from life events as a mental predisposition factor separate from emotion dispositions as it is more directly tied to eliciting events. The goal was to examine, in an exploratory manner, the theoretical framework outlined earlier with respect to direct effects and mediation effects in a large sample of the population. Given the aim of identifying particular risk factors for emotion disorders, it is of particular importance to rely on panel data in order to have access to a large, representative, randomized sample living in a specific country.

### Materials and Methods

#### Participants

The current analysis is based on a representative population sample of the SHP, a unique longitudinal survey in the social sciences interviewing all household members of a random sample of private households in Switzerland since 1999 (swisspanel.ch; Tillmann et al., 2016). The principal aim of this annual household panel survey is to observe social change, in particular the dynamics of changing living conditions in the resident population of Switzerland (Tillmann et al., 2021). The SHP survey is a general survey that covers a wide range of areas in the social sciences—for each individual participant, the SHP provides information on socio-demographic characteristics, household and family composition, health and quality of life, experience of major life events, employment status and income, social networks and integration, political orientation and values, degree of subjective well-being, and self-perception (e.g., self-esteem, personality and emotionality).

The sample used for this study (Wave 14, SHP 2012) consisted of 4,859 individuals (2,188 male and 2,671 female) between 18 and 65 years old. The majority of the participants in the sample were married, had a middle level of education, and were active in the labor market.

In accordance with international ethical standards of research on humans, the SHP was approved by the Research Commission of the University of Neuchâtel (Zimmermann-Sloutskis et al., 2010). During every data collection wave, each household in the panel receives a letter containing extensive information about the SHP and its aims. Moreover, the length and content of the interviews are specified and the confidentiality, anonymity, and exclusive use of the data for scientific research purposes are explained in detail. Shortly afterward, all households are contacted *via* telephone by the survey institute that conducts the data collection, and each household member can freely consent to participation or refuse it (Zimmermann-Sloutskis et al., 2010).

#### Assessment Instruments

From the large number of items in the survey questionnaire, and on the basis of theoretical considerations and past reports in the literature, we selected those variables that were of major importance for the issue to be investigated. This section is organized by the different types of these variables, describing the SHP questions used to measure the variables.

##### Risk for Mood Disorder

The central dependent variable in this study was the frequency with which participants experienced episodes of depression or anxiety severe enough to be considered a potential risk factor for clinically relevant mood disorders. The most widely used clinical screening instruments worldwide are part of the Patient Health Questionnaire (PHQ) family (Kroenke et al., 2010). Specifically, the PHQ-2 is a validated ultra-brief screener that comprises the first two items of the full PHQ-9 scale (depressed mood and anhedonia), which are core criteria for depressive disorders (Kroenke et al., 2003). Two questionnaire items in the health section of the SHP survey closely reflect the meaning of these two PHQ-2 items: “Do you often have negative feelings such as having the blues, being desperate, suffering from anxiety or depression?” for depressed mood and “How often do you feel you have plenty of strength, energy and optimism?,” which was inverted to reflect anhedonia (the translations of these central items into the three main national languages spoken in Switzerland are shown in Supplementary Table S2). In line with the general response structure in the SHP, both items were rated on a 10-point scale from *never* to *always*. An indicator of risk for mood disorder has been constructed from these two items by computing the mean (with an inverted score for the strength, energy, and optimism item). This dependent variable is referred to below as *Prevalence Depression/Anxiety*, referring to the imbalance between the two types of experiences.

This indicator combines several symptoms of potential emotional disturbances that are frequently assessed in separate questions. However, it would be problematic to ask separate questions for several different syndromes in a large panel survey because not only does each item count in a massive survey, but it is also not obvious that laypeople make the same fine distinctions as clinicians. Furthermore, it is a well-established fact in the clinical literature that there is a high degree of comorbidity of anxiety and depression, each constituting a major risk factor for the other (Gorman, 1996; Pollack, 2005; Cummings et al., 2014). Jacobson and Newman (2017) conducted a systematic review and meta-analysis of 66 studies involving 88,336 persons that examined the prospective relationship between anxiety and depression at both symptom and disorder levels. It showed that all types of anxiety symptoms predicted later depressive symptoms and that all types of depressive symptoms predicted later anxiety symptoms. This close link also exists in subclinical populations, as demonstrated by the high correlation between the two standard depression and anxiety inventories, the PHQ and the Generalized Anxiety Disorder scale (GAD; Kroenke et al., 2010, Teymoori et al., 2020).

##### Mental Predispositions

This class of variables refers to an individual difference factor consisting of a certain readiness to react in a specific manner to events, conditions, and situations that affect the individual. As mentioned in the introduction, this research is based on a theoretical framework that gives prominence to *emotion dispositions*: the tendency, in part due to specific appraisal biases, to experience certain emotions in encountering comparable events more frequently and more intensively than other individuals do. For the specific issue of determining risk factors for depression and anxiety disorders, we also considered a second type of mental predisposition, referred to as *Severity of suffering*: a stable tendency to suffer more frequently and more intensively, compared with other individuals, for comparable life events such as pain (Bustan et al., 2018), health concerns (Williams and Wiebe, 2000; Hettema et al., 2006; Hammen, 2018), major life events such as bereavement (Stroebe and Schut, 2001), or other serious worries (Wang, 2005), which were rated separately.

##### Emotion Dispositions

The survey questionnaire contains a special section, devoted to everyday experiences, with separate items for four frequently occurring emotions (Scherer et al., 2004): “How frequently do you generally experience the following emotions: joy - anger - sadness - worry?” rated on a 10-point scale where “0” means *never* and “10” means *always*. In view of the aim to identify risk factors for depression and anxiety disorders, we focused on potential dispositions to experience worry and sadness *more frequently than other emotions*. One possibility is to use a positive-negative affect imbalance variable. The utility of a relational measure of affectivity has been clearly demonstrated in the literature. Thus, Watson et al. (1988) demonstrated the diagnostic importance of considering both positive and negative affectivity in relation to anxiety and depressive disorders. The states-of-mind model (Schwartz and Garamoni, 1989) emphasizes the *balance* between positivity and negativity, subsequent research showing its clinical relevance for depression (Garamoni et al., 1991). More recently, Cheng and Furnham (2003) reported a significant correlation (*r* = 0.56) between an affect balance scale and the Beck Depression Inventory score. The notion of “affect balance” is also used in well-being research: people with higher scores have a positive affect that strongly “outweighs” the experience of negative affect (Diener et al., 2010). Dejonckheere et al. (2018) confirmed that the nature of the relation between positive and negative affect in a person’s emotional experience is indicative of psychological well-being, in particular the experience of depressive symptoms, typically characterized by diminished positive affect (anhedonia) and increased negative affect (depressed mood). Veilleux et al. (2020) recently showed the utility of affect balance as a predictor of emotions experienced during daily life, finding that people higher in affect balance report significantly more positive than negative affect on a daily basis.

However, in the interest of consistency across studies, we computed a composite variable, *prevalence of sadness and worry*, by calculating the mean of the frequency ratings for worry and sadness (reliability: Cronbach’s alpha = 0.75).

##### Severity of Suffering

Although suffering involves a high degree of emotionality, it is different from clearly defined emotion episodes triggered by specific events in that it reflects a persistent, conscious concern with specific problems such as health-related pain, enduring effects of life events such as bereavement, and continuous worry about central issues such as work life and financial resources. We assume that high ratings on the degree of suffering reflects individual differences in the susceptibility to suffer comparatively more severely than others under comparable conditions.

We used the mean of the following three variables from detailed questions in the SHP questionnaire: satisfaction with state of health (inverted scale); average suffering from major life events (such as bereavement); and average degree of worrying over major issues, such as work, finance, and insecure self-esteem, as the relevance of these as risk factors is well established (see Wang, 2005; Orth et al., 2009, 2016; Rieger et al., 2016; Feng et al., 2019). The reliability of the combined score is acceptable, with Cronbach’s alpha = 0.69.

##### Appraisal Bias

To assess the theoretical construct for the appraisal criterion of coping potential, we used a number of items in the survey questionnaire that are intended to measure control and coping beliefs, as well as self-esteem, many of which arguably reflect a potential appraisal bias. These questions were derived from two well-established scales (Pearlin and Schooler, 1978; Lachman and Weaver, 1998). Using confirmatory factor analyses (CFA), we identified the following five subscales of a “sense of control and coping potential” construct: Determination, Self-efficacy, Agency, Power, and Self-esteem. This model shows good to excellent factor loadings and a good model fit (see Appendix 1 in the online supplemental materials for further details and references).

A principal component analysis of these five subscales resulted in the extraction of a single factor (with Eigenvalue >1). Table 1 shows the component matrix. In the interest of obtaining a clearly defined one-dimensional predisposition variable, we computed the mean of the subscales loading highly (>0.5) on this single factor, the determination, self-esteem, and power subscales, to create a composite appraisal bias variable of *low self-perceived control/coping potential* (inverted scale). For the sake of readability, we abbreviate this variable as *low coping potential* in the remainder of this article. A reliability analysis yielded a Cronbach’s alpha of 0.66 for the mean of three combined subscale variables (based on seven individual items), indicating an acceptable level of reliability.

**Table 1:** Component matrix resulting from a principal component analysis of five control subscales.

	Component
	1
Control: determination	0.797
Control: self-esteem	0.717
Control: power	0.690
Control: self-efficacy	0.497
Control: lack of control	−0.484

##### Background Variables

In addition, we included a number of questionnaire variables that, judging from the literature, are potential predictors for appraisal bias, emotion disposition, and risk for disorders: *personality* (extraversion, neuroticism; as measured by the Big Five Inventory-10; Rammstedt and John, 2007), *motivation* (achievement, social fulfillment; based on ratings of five important things in life, adapted from an early version of the Aspects of Identity Questionnaire; see Cheek and Cheek, 2018), *past experience* (number of major life events last year), and *socio-demographic variables* (gender: male/female, age, education level, and language spoken in the respective household: Swiss German, French, Italian). For each category, we included those items found to be pertinent in past research in this area. Language spoken was chosen as an important biographic category, given the multilingual background of Switzerland and the concomitant cultural differences.

#### Procedure

The SHP questionnaire is administered *via* a computer-assisted telephone interview (CATI) survey on a yearly basis. The current data set was obtained from Wave 14 of the SHP survey, collected in 2012. All of the statistical analyses reported in this article were performed by the authors from the original SHP panel data.

#### Statistical Analysis

Our aim was to examine the hypothesis that the risk for frequent feelings typical of depression and anxiety is increased by appraisal biases, in particular, an underestimation of one’s coping potential, and likely to be mediated by a disposition to more frequently experience the emotions of sadness and worry, as well as to suffer more severely from health problems, major life events, and serious worries. In addition, we wanted to examine the way in which these central variables are affected by a number of background factors (see Measures section above).

The procedure chosen for this purpose was a theory-based path model, as illustrated in Figure 1, which presumes a causal chain from more distal to more proximal causal factors acting on the dependent variable. The basic assumption is that the more proximal factors have a stronger impact on the dependent variable (explaining a larger amount of variance). The impact of more distal factors is mediated by the more proximal intermediate factors, but can also have, in addition, a direct, non-mediated impact on the dependent variable (a more detailed description of such causal path models can be found in Bänziger et al., 2015). One possibility to statistically test the goodness of fit of such a theoretical path model is to use a hierarchical multivariate regression analysis with stepwise selection of predictors at each level of the hierarchy (see also Cheng and Furnham, 2003; Poppe et al., 2012, and Scherer, 2021, for further examples of using theory-based hierarchical regression analysis).

As shown in Figure 1, in the first regression (R1), the dependent variable (Depression/Anxiety) is regressed onto the hypothetically most proximal predictors, emotion disposition (Sadness/Worry) and the appraisal bias variable (Low coping potential) as the next most proximate predictor, and, finally, on the different classes of background variables, in this order. The hierarchical regressions were performed with SPSS, in which the various predictors were added in subsequent blocks by using the STEPWISE entry procedure (using *p* < 0.001 as the entry and *p* > 0.005 as the removal criterion). In the next regressions (R2), emotion disposition (Sadness/Worry) and severity of suffering were treated as the dependent variable and regressed onto the appraisal bias variable (Low coping potential) and onto the different classes of background variables, in this order. Finally, the appraisal bias variable (Low coping potential) was regressed onto the different classes of background variables.

In addition, we used discriminant analysis to obtain an estimate of the degree to which the central mediating variables (coping appraisal bias and emotion disposition toward sadness/worry) allow us to classify individuals likely to be at risk for mood disorder. For this purpose, we classified the sample on the basis of the percentiles in the distribution of the Risk for Mood Disorders variable, low risk being defined as belonging to the lowest 25% of cases, medium risk for the range from 25% to 75%, and high risk for the upper 25% of the distribution. The SPSS version 25 package was used for all analyses.

#### Transparency and Openness

The sample size reported above was determined by the total number of cases in Wave 14 of the annual SHP survey. Depending on the specific analyses, cases with missing data for the respective variables were automatically excluded (minimal *N* = 4,476). All manipulations and all measures in the study are described in the Materials and Methods section. Data were analyzed with SPSS, version 25. The SHP is financed by the Swiss National Science Foundation. The SHP data are available upon application to SwissUbase. (https://www.swissubase.ch/en/catalogue/studies/6097/16588/overview). The SHP data are freely available to scientists who sign a contract agreeing to legal and ethical conditions that apply to the use of the data for research purposes (Tillmann et al., 2016). The Swiss Centre of Expertise in the Social Sciences (FORS) oversees the SHP. This study’s design and its analysis were not preregistered.

### Results

The correlation matrix and the detailed results of the hierarchical regression analyses are shown in Supplementary Tables S3 (correlation matrix) and S4 (beta coefficients). Table 2 shows a summary of the results for regression steps R1–R3. The variance accounted for in the dependent (criterion) variable (adjusted *R*^2^) is due to both *direct* effects from the predictor classes and *mediated* effects *via* the intermediate variables in the path model shown in Figure 1. Of the extracted prediction solutions, we chose the one after which the *R*^2^ change had a value of <0.001. Table 2 also shows the standardized beta coefficients for the variables entered into the equation at the chosen level, reflecting the strength of impact for the respective predictor. Supplementary Figure A1 shows a detailed path diagram of the results based on the theoretical predictions.

**Table 2:** Summary of the results of the hierarchical regression analyses (stepwise entry) according to the theoretical ordering (in regression steps R1–R3) shown in Figure 1 (listing adjusted *R*^2^ and beta coefficients).

*Predicted*	Risk for mood disorder	Sadness/worry disposition	Low coping potential
Adjusted *R*^2^	0.48	0.39	0.34
Direct effects	R1	R2	R3
***Predictors***			
Sadness/worry disposition	0.28	*	*
Low coping potential	0.26	0.19	*
*Background factors*			
Gender	0.05	0.09	-
Age	-	0.08	0.07
Education	-	0.06	-
Language region	0.10	0.28	0.05
Neuroticism	0.13	0.15	0.15
Extraversion	-	−0.09	−0.13
Achievement motivation	-	-	−0.22
Suffering from life events	0.27	0.28	0.41

Due to the hierarchical nature of the regression steps, some variables are predictors or predicted variables, depending on the step; * not entered; -, coefficient not significant.

As risk analyses are often based on categorization, we also computed a discriminant analysis using stepwise entry of the most powerful predictor variables with prior probabilities for each of three groups (low, medium, and high risk) set to chance level at 33.3%. The analysis produced two canonical discriminant functions, with Function 1 explaining 99.8% of the variance. Variables were entered in the following order: (1) emotion disposition toward sadness/worry, (2) low coping potential appraisal bias, and (3) severity of suffering. A classification table with the cross-validated classification result matrix, shows a correct classification rate of 69.4% for the high-risk group. The same analysis was performed by using only the appraisal bias and emotion disposition variables as predictors. The results, shown in Table 3, are similar, with a correct classification rate of 66.9%, indicating that severity of suffering adds little if any predictive power after the effect of appraisal bias and emotion disposition is accounted for. Using appraisal bias as the only predictor yields a correct rate of 59.9%.

**Table 3:** Classification table with cross-validated results of a stepwise discriminant analysis of the categorized risk for mood disorder variable.

	Risk group	Low	Medium	High	Total
Count	1 Low	1,231	428	157	1,816
	2 Medium	616	699	445	1,760
	3 High	132	293	858	1,283
Percentage	1 Low	67.8	23.6	8.6	100.0
	2 Medium	35.1	39.7	25.3	100.0
	3 High	10.3	22.8	66.9	100.0

57.4% of cross-validated grouped cases were correctly classified; stepping criteria: entry (*F* > 3.84), removal (*F* < 2.71).

### Discussion

We predicted that an appraisal bias of low coping potential would be associated with an emotion disposition of sadness/worry prevalence and with severity of suffering, both of which, in turn, would be associated with a clinically relevant risk for depression/anxiety disorders. This is exactly what we find. There is also a direct effect from appraisal bias to risk for depression/anxiety disorders, indicating that mental predispositions do not completely mediate the effect, but that an appraisal bias of low coping potential has an additional *direct* impact (as shown by the beta weight of 0.26). Moreover, there is a direct, non-mediated effect from neuroticism (which may be partly due to the similarity of one of the two items indexing neuroticism in the Big-Five-Inventory with the anxiety experience item). These direct effects (R1) are plausible, given the reports on the importance of these factors for depression and anxiety described in the literature (Kotov et al., 2010; Paulus et al., 2016). Notably, the percentage of variance accounted for in the risk for depression/anxiety disorders is extremely high for the large sample size (adjusted *R*^2^ = 0.48).

Several variables contribute to the sizable percentage of the variance accounted for in the case of the sadness/worry emotion disposition (adjusted *R*^2^ = 0.39), the most important being, as hypothesized by the appraisal bias theory described in the introduction, a low coping appraisal bias. Various background variables also have a number of interesting effects. As one would expect, one of these variables is neuroticism, given that experiences of sadness and anxiety are in fact part of the definition of this personality trait variable, as frequently reported in empirical research (Williams and Wiebe, 2000; Kotov et al., 2010; Sutton et al., 2011; Paulus et al., 2016; Hong and Tan, 2020). In contrast, the strong effect of language spoken in the household on feelings of worry and sadness may seem somewhat surprising at first sight. A similar effect was found in the Swiss semi-representative study mentioned earlier (Scherer et al., 2004). So far, there is no obvious explanation for this finding.

In turn, low coping potential appraisal bias (adjusted *R*^2^ = 0.34) is, rather plausibly, predicted by neuroticism and the severity of suffering from life events experienced last year. Achievement motivation and extraversion have a negative effect, which is also to be expected (Verduyn and Brans, 2012).

The classification results of the discriminant analysis (Table 3) are rather impressive, given the large *N*. For this analysis, we used a categorized risk for the mood disorder variable (low, medium, high risk) that was based on the mediating variables (low coping appraisal bias and sadness/worry emotion disposition). Both the high- and the low-risk groups are classified correctly in approximately 70% of all cases (more than double the chance level of 33.3%), suggesting that the measurement of these variables in large surveys can be an important asset in screening for risk factors involving major mood disorders. This is an interesting option for screening large groups of the population, as the questions concerning these three variables, compared with direct questions about the symptoms of disorder, are likely to be less subject to potential defense mechanisms against disorder implications of the respondents.

#### Limitations

As is the case with all field studies, there are some limitations to the current study. In a nationwide household panel survey, only self-report can be obtained, and only correlational statistics can be used. The advantage of using large-scale panel data is that we can work with (1) a random sample representative of the general population and (2) a sample size of several thousands of participants. However, the design of such a panel—covering a broad range of domains and topics of interest to the social sciences—precludes the use of multi-item inventories (Eisinga et al., 2013) and privileges short, validated scales or single-item scales. In consequence, the SHP survey questionnaire includes only a single item to measure the frequency of experiencing states of despondency, desperation, depression, and anxiety. Apart from the methodological constraints, this approach is justified by the high degree of comorbidity between depression and anxiety (and the resulting similarity of risk factors and symptoms; see the meta-analysis by Jacobson and Newman, 2017).

It should be kept in mind that a fair number of studies have shown that one-item tests perform well in comparison to multi-item questionnaires and are thus recommended for cases in which the use of long questionnaires is impossible (Mahoney et al., 1994; Spitzer et al., 1994; Whooley et al., 1997; Davey et al., 2007). Clearly, a general nationwide household survey, covering a large number of domains, constitutes such a special case. Furthermore, if a one-item measure shows a high-power effect with a large number of observations, generating low value of *p*, there is little need to regret the lack of reliability measures. This is especially true because scale items are often highly redundant and are perceived as such even more in a CATI survey such as the SHP. It should be noted that in this case, we also used the second item (corresponding to an equivalent item in the PHQ-2) to construct a composite variable for depression/anxiety prevalence.

A further limitation is the assessment of appraisal bias by using items taken from well-established coping belief scales and emotion dispositions *via* questions on the frequency of experiencing major emotions. A more appropriate approach would be the measurement of both appraisal biases and emotion disposition with the help of scenario-based instruments, using an appraisal bias and emotion disposition scale such as the Emotion Disposition Index (EmoDis; Scherer, 2021). However, apart from the fact that this instrument was only recently developed, it would not be feasible to integrate even a short version of it into a major, nationally representative, longitudinal household survey. In consequence, for the moment, the approximate assessment *via* validated questionnaire items is unavoidable in this context.

Finally, the causal assumptions underlying the theoretical model presented in Figure 1 cannot be confirmed by the analyses of a single time window. Although the results reported here are very much in line with these theoretical predictions, they can only strengthen the plausibility of these models, the issue of causality requiring further examination, preferably by studies that use time series. In addition, we would expect recursive relationships between appraisal bias, emotion disposition, and mood disorders.

## Study 2

Given the limitations of a nationwide representative household survey, we wanted to examine the predictions of the appraisal bias/emotion disposition model presented in the introduction, using established instruments in a specially recruited sample of participants. We chose young adolescents as the target population, as there have been several reports of the high incidence of depression and general anxiety in younger adults (Mojtabai et al., 2016; Hawes et al., 2021; see also https://www.who.int/news-room/fact-sheets/detail/adolescent-mental-health).

To measure the risk for developing these emotion disorders, we chose two of the most widely used instruments in this domain, the PHQ-8 (Kroenke et al., 2009) and the GAD-7 (Spitzer et al., 2006). Appraisal bias and resulting emotion dispositions are measured by two new instruments, the Emotion Index (EI), measuring the self-reported frequency of different types of emotional experiences, and the Emotion Disposition Index (EmoDis), measuring the respondent’s projected appraisal pattern and emotional reaction in a number of typical emotion-eliciting scenarios. These instruments were recently validated in two large-scale studies cited earlier (Scherer, 2021): (1) The EmoDis was administered to several thousand adults from many cultural backgrounds in personnel assessment contexts (*N* = 3,012), demonstrating the existence and intensity of emotion dispositions, as well as identifying potential individual difference correlates; and (2) the EmoDis was used in a representative survey panel study of US citizens (*N* = 190), assessing both appraisal biases and emotion dispositions, allowing an in-depth analysis of their relationships (in addition to examining the effect of correlates).

There are two major questions to be investigated in this study: (1) Can the important effect of specific appraisal biases on emotion dispositions be replicated in a sample of participants drawn from another culture and language, and (2) can the effects of appraisal bias and emotion dispositions on the risk for depression and generalized anxiety, found in Study 1, be replicated in a study with a group of young adults by using validated assessment scales in a web-based survey?

As to (1), Scherer (2021) investigated the following predictions on the effect of appraisal biases on emotion disposition (see also Supplementary Table S1):

- low relevance bias > indifference (good humor)
- obstructiveness (negative valence) bias > dissatisfaction (generally negative emotions)
- external attribution bias (other agency) > anger, contempt
- internal attribution bias (self-agency) > guilt/shame
- control/power underestimation (coping potential) bias > sadness, worry
- high norm compliance bias (applied to internal causal attribution) > guilt/shame.

Using the same EmoDis instrument in the current study, we will investigate these same hypotheses and compare the results to those reported by Scherer (2021).

As to (2), the predictions on the effects of appraisal biases and emotion dispositions on potential risk factors for emotional disorders, published in Scherer (2021), are shown in Supplementary Table S1. Of particular importance for the replication of the results in Study 1 is the prediction concerning risk for depression and anxiety, considered to be caused by an underestimation bias for control and power and frequent experiences of pessimism, sadness, and worry. To test these predictions, we used both self-report measures and the scenario-based instrument EmoDis.

### Materials and Methods

#### Participants

A total of 152 adults (Italian university students, mostly from psychology, 67% female, mean age 24.8 years, *SD* 6.95) were recruited on a voluntary basis (without payment) for a study on patterns of emotional experience. Informed consent was acquired from each participant through the Qualtrics online platform. The study design and procedure were approved by the Ethics Committee of the University of Bologna.

#### Assessment Instruments

The risk for depression/anxiety disorder was measured by administering the PHQ-8 and the GAD-7. Both tests list specific symptoms (seven or eight) and ask for the relative frequency of their occurrence. The results of these tests are generally interpreted by defined cutoff points after which the person is considered to be at different degrees of risk for developing a clinical disorder. Given the high comorbidity of anxiety and depression (Gorman, 1996; Pollack, 2005; Cummings et al., 2014; see the discussion under the Measures section for Study 1), we computed the mean of the two separate test scores as a combined risk estimate for depression/anxiety disorder. This is also justified by the high correlation between the two separate scores in the current study (*r* = 0.73, *p* < 0.01). We used the raw score for the combined variable to benefit from higher resolution in the multivariate analyses.

To measure appraisal bias and emotion dispositions, we used both self-report and a scenario approach, specifically the EmoDis assessment instrument (Scherer, 2021), which consists of several scenarios (situation descriptions) representing typical emotion-eliciting situations. Participants are asked to imagine experiencing the respective situations and to answer the following questions on how they appraised the situation and which emotion(s) they would probably experience in this case:

##### Appraisal

The event is

not very important for me    very important for me (*relevance*)

negative for me        positive for me (*valence*)

difficult for me to deal with     easy for me to deal with (*coping*)

morally questionable       morally acceptable (*norm compatibility*)

caused mainly by myself       caused mainly by others (*agency*)

(using a -100-0-100 scale with a “not pertinent” option)

##### Emotional Reaction

How intensely would you experience each of the following emotions?

indifferent

anxious/worried

sad/dejected

annoyed/angry

ashamed/guilt feelings

(on a *not at all* to *very much* 0–100 scale)

The nine standard scenarios used in this study are listed in Appendix 3 of the online supplemental materials. Given the length of the overall assessment session, a brief version of the EmoDis with four of the nine scenarios (numbers 2, 4, 5, and 7, bolded in Appendix 3) are currently used in further research in this area. As the scores for the nine-scenario and the four-scenario versions of the scale correlate with *r* = 0.8–0.9, and as the data are averaged over scenarios, we used the four-scenario version of the scale in the present report to facilitate future comparisons of results.

We used the EmoDis described earlier as a direct self-report indicator of emotion disposition to assess the frequency of specific emotional experiences. Participants were asked to report the frequency of having experienced during the last 2 weeks each of the following emotions: joyful/happy, anxious/worried, annoyed/angry, sad/dejected, ashamed/guilt feelings, bored/disinterested. Of particular interest were the responses on the sad/dejected and anxious/worried items. Given the high correlations between these two emotions (*r* = 0.57, *p* > 0.01) and the evidence of a strong comorbidity between these disorders in the clinical literature (see review in the Measures section of Study 1), as in Study 1 we constructed a combined sad/worry score (by using the mean value of these measures).

To obtain a brief self-report indicator of appraisal bias, as in Study 1, we used a subset of four items from established control belief and self-efficacy scales (see Appendix 1 and 2 in the online supplemental materials for further details) to assess the perceived ability to control events and cope with consequences. A reliability analysis yielded a Cronbach’s alpha of 0.66 for these four items.

Background variables were assessed by the usual questions concerning gender, age, level of education, occupation, and country of birth. Given the homogeneity of the student sample, only the Gender variable was used in the analyses. Respondents were also asked how often they had suffered from different unpleasant life events in the last 3 months (never, once, twice, three times).

#### Procedure

All the scales were administered online through the Qualtrics platform, providing target students with a specific anonymous web address that directly connected the participant with the survey. After an introduction to the study, the participant was asked to accept informed consent in order to proceed. Demographics questions were presented first, followed by the different scales presented in randomized order between participants. Respondents were allowed to finish the survey within 24 h from first access. The mean duration of the response times was 30.6 min. Prevent indexing was activated to block search engines from including the survey in the search results. Multiple submissions were also prevented by placing a cookie on the respondent’s browser. The survey was completely anonymous and no personal data were recorded.

#### Statistical Analysis

Given the explicit causal assumptions of the underlying theoretical framework, as in Study 1 (and in Scherer, 2021), we used hypothesis-guided path analysis, illustrated in Figure 1, to examine the effect of the different classes of predictors on the risk for emotional disorder. The path analyses were computed *via* multivariate hierarchical regression analysis (using the GROUP function in the SPSS-25 regression command). For a more detailed justification of this procedure, see Scherer (2021) and Appendix 1 and 2 of the online supplemental materials.

#### Transparency and Openness

A sample size of *N* > 150 had been targeted. Only submissions with a duration greater than 10 min and with a progress rate of 100% were included in the analyses, filtering out incomplete or inaccurate surveys. All manipulations and all measures in the study are described in the Materials and Methods section. Data were analyzed by using SPSS, version 25. The data files are available from the corresponding author upon reasonable request.

### Results

Regarding the first issue to be examined, the effects of appraisal bias on emotion dispositions, the results based on a stepwise hierarchical regression analysis are shown in Table 4 (columns 2 and 4). To facilitate interpretation of the statistical results, we compared them with results obtained in a similar survey study that used the same instruments (Qualtrics) with a representative sample of US citizens (columns 4 and 5; see Scherer, 2021). For both samples, Table 4 shows the results of regressing appraisal bias (EmoDis and LowControl scale) and background variables (gender, suffering) on the different emotion dispositions, listing the adjusted *R*^2^s for the set of predictor variables entered into the equation and the respective beta coefficients.

**Table 4:** Study 2: hierarchical stepwise linear regressions of appraisal ratings, low control/power beliefs, and background variables on emotion ratings.

Sample	Italian sample		United States panel (Scherer, 2021)	
Participants	*N* = 152 young adults/students, mean age 24.8, 67% female		*N* = 190 adults; mean age 45.5, 49.2% female	
**Emotion**	**Predictors and beta coefficients**	**Adj** *R* ^**2**^	**Predictors and beta coefficients**	**Adj** *R* ^**2**^
*Annoyed/angry*	Valence −0.23, low control 0.18; norm compliance −0.15, gender −0.18	0.13	Coping −0.31, other agency 0.30	0.21
*Anxious/worried*	Coping −0.38, relevance 0.21	0.22	Coping −0.39, valence −0.18, age 0.13	0.30
*Sad/dejected*	Coping −0.35, relevance 0.23, low control 0.22	0.30	Coping −0.35, valence −0.25	0.27
*Ashamed/guilt feelings*	Coping −0.29, other agency −0.20, low control 0.22	0.18	Gender −0.18	0.06
*Indifferent (good humor in US sample)*	Valence 0.16, gender −0.17	0.13	Valence −0.10, coping 0.39, relevance −0.34, norm compliance 0.26	0.43

Enter criterion 0.05; exit criterion 0.10.

The data obtained with the Italian sample very closely replicate the results of the US panel study in the case of anxiety and sadness, even to the point of agreement on the strength of the effect (beta coefficients). This result also confirms the theoretical prediction of a coping potential bias (control/power underestimation) as a probable causal factor. There is a difference in terms of the appraisal of the importance of the eliciting event: Whereas the US participants highlighted the negative valence of the event, the Italian participants underlined the high relevance of the event. The latter seems somewhat more appropriate in terms of the discrimination of the emotions, as all of the scenarios depicted events with mostly negative consequences.

There is also partial agreement for anger, as the low control/coping capacity was invoked (in the case of the Italian sample only *via* the self-report measure). Apart from this, the US participants highlighted the agency of another person as being essential (as theoretically predicted), whereas the Italian participants saw a larger effect in the negative valence of the event and the norm violation involved (which might be linked to other agency). Male Italian participants seemed to have a more pronounced anger disposition.

For shame/guilt, whereas there were no significant appraisal bias effects for the US sample, for the Italian participants, as theoretically predicted, an internalization bias in causal attribution (self-agency) increased the likelihood of experiencing shame/guilt. For this sample, low control/coping capacity was also an important factor.

There was a difference for Good humor/indifference: Whereas US participants reporting this feeling more frequently seemed to have an appraisal bias of downplaying relevance and potential norm violation, they boosted the appraisal of their coping potential. In the Italian sample, indifference was more frequent for males with a positive valence bias.

On the whole, with respect to the first aim of Study 2, these results largely confirm the theoretical predictions on the relationships between appraisal bias and emotion dispositions and the high likelihood of the generalizability of the predicted effects across samples from different cultures.

The second issue to be investigated, based on the results in Study 1 described earlier, concerns the extent to which an emotion disposition toward frequently experiencing sadness, anxiety, and worry (presumed to be caused in part by appraisal biases), may contribute to the risk of developing mood disorders, specifically depression and generalized anxiety. The dependent variable measuring risk is the mean score of the PHQ-8 and the GAD-7. As described in the Materials and Methods section, the central predictor variables were measured by two types of assessment for appraisal bias and emotion dispositions, one *via* direct self-report (the Control Belief Questionnaire and the EmoDis), the other *via* an indirect approach (EmoDis), asking participants to imagine the experience of a number of emotion-eliciting scenarios and consequently rating the most likely evaluation patterns (*via* appraisal checks) and the most likely emotional response. Here these variables are examined with respect to their predictive validity for the mood disorders.

**Figure 2:**
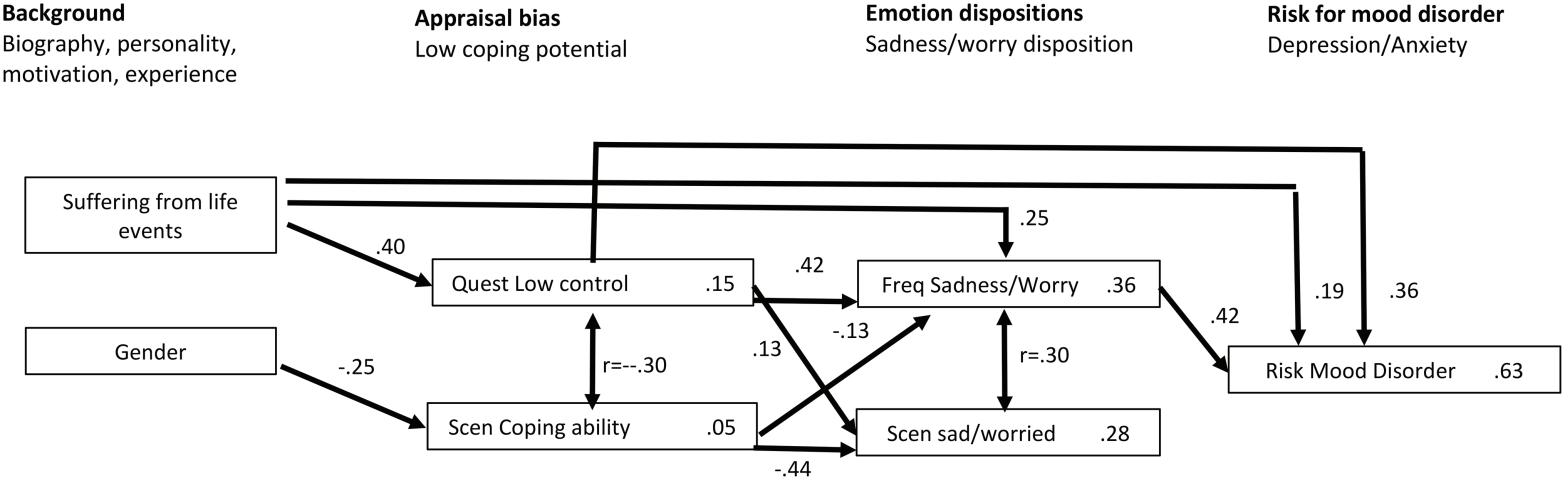
Path diagram showing the combined results (significant predictors) of three subsequent hierarchical regression analyses: (1) dependent variable (depression/anxiety risk factor for mood disorder) on sadness/worry emotion disposition, appraisal bias, and background variables; (2) “Sadness/Worry” emotion disposition variables on appraisal bias and background variables; and (3) appraisal bias variables “Control” and “Coping Potential” on background variables. Path coefficients (beta) on arrows, Adj. adjusted R2 in boxes; risk for mood disorder = mean GAD/PHQ, Scen—Scenario measurements (appraisal, emotions, Freq—last 2 week frequency, Quest—control belief questionnaire; arrows shown only for beta coefficients with *p* > = 0.10.

The results of the stepwise regression analyses are shown in Figure 2. Overall, these results provide further supportive evidence for the appraisal bias/emotion disposition model of emotional disorder. As theoretically predicted, indicators of low control/coping appraisal predict the frequent experience of sadness and worry, and, in turn, the latter emotion disposition contributes to the risk for mood disorders.

A more detailed analysis of the path diagram shown in the figure reveals a number of important issues. One important question concerns the utility of using an indirect approach to assess appraisal bias and emotional dispositions. There are significant relationships between the respective variables and those obtained by direct self-report of frequency of experience (labeled Freq) suggest that the scenario measures capture some of the important variance in appraisal and emotional reactions. However, the missing arrow between the scenario measurement (labeled Scen) of sad/worried and risk for mood disorder could be interpreted as scenario measures not being very good predictors of emotional disorder. Yet, there is a significant correlation of *r* = 0.30 (*p* < 0.01) between these two variables. The reason that this does not produce a significant beta weight in the regression is because in the stepwise procedure, only additional variance counts—in this case, the stronger effects of the self-report emotion disposition measure already represent most of the variance due the relationship between emotion disposition and risk. This raises the important issue of method effects. After all, the PHQ and the GAD are based on direct self-report, as is the Control Belief Questionnaire and the EmoDis.

One surprising result is the strong effect of the reported suffering from unpleasant experiences in the last 3 months on all of the variables, including a direct, unmediated effect on emotional disorder. However, again, the degree of suffering is based on direct self-report and may thus also benefit from the method effect—a tendency to report more negative emotional experiences than others. This illustrates a major dilemma for both emotion and psychopathology research, given that self-report assessment is a mainstay for the methodology in this area and that there are few established methods for statistically controlling for method factors.

One way to examine the added value of scenario measures is to compare two subgroups of participants with high self-report sadness/worry frequency over 2 weeks, one that has particularly high values for the scenario sad/worried variable and one with lower levels (*N* = 73). To test this hunch, we computed *z*-scores and determined the group with *z* > 0 for the frequency measure (*N* = 94 of the 152 participants). This group was then split into two subgroups by using *z* = 1 for the scenario measure as a cutoff point to select high scorers. We found the following means on the PHQ/GAD risk variable: above the cutoff 20.8 (*N* = 21) and below 18.5 (*N* = 73). A one-way analysis of variance showed that the difference is significant with *p* = 0.037. Thus, it seems that using the indirect scenario method does in fact improve the detection of serious risk levels.

### Discussion

#### Limitations

As in most empirical research, there are a number of limitations inherent in the methods chosen. Thus, in Study 1, the SHP has some limitations in terms of the equal representation of subgroups, but it is based on a very large random sample of individuals. Furthermore, the CATI survey format limits the number of items to measure central concepts and privileges direct self-report. Study 2 is based on an Italian student sample (which, however, yields results that are highly similar to those obtained in a more representative survey).

As briefly discussed earlier, one central constraint for a major segment of the research on emotion and emotion disorder is the need to rely on self-report, as there are no other, more objective measures, to assess subjective feeling states. However, in Study 2 we added the indirect scenario assessment to complement the assessment procedure and found reasonably high correlations with the questionnaire instruments. Arguably, this approach is highly promising, given that it focusses on the central phenomenon—emotion—and can be used repetitively, on a daily or weekly basis, to obtain information about dynamic mood changes over time (see Kuppens et al., 2010). It may also be particularly useful for younger participants with a relatively less stable self-image and fewer life experiences.

## Overall Conclusions

In this article, based on two complementary samples, one random nationwide sample and one selected sample based on a student population, we attempt to demonstrate the well-foundedness of a nomological network of interrelated variables based on a theory of emotional functioning—appraisal theory—that is generally considered the most comprehensive attempt to map out this domain (Moors et al., 2013). In particular, we hope to build bridges between the communities of researchers studying emotion, personality, and individual differences on the one hand, and clinical psychologists and psychiatrists on the other, by demonstrating the utility of using theories and findings on “normal” emotionality in order to better understand abnormal affective disorders (Scherer and Mehu, 2015). In this way, we hope to contribute to the development of an emotion-focused clinical approach in the spirit of L. S. Greenberg's (2019) theory of functioning.

We consider the results of the two studies reported here, summarized in Table 2 for Study 1 and Figure 2 for Study 2, to provide important empirical evidence for appraisal bias theory according to which habitual biases in event evaluation can potentially lead to stable emotion dispositions, which, in some cases, may develop into mood disorders, in particular depression and generalized anxiety. Our findings confirm and extend a recent empirical study that demonstrated the close link between habitual underestimation of one’s coping potential and emotion dispositions such as worry and sadness (Scherer, 2021). We were also able to examine the effect of some background factors. Although age and gender have little effect in our samples, the number of life events and the degree of suffering caused is clearly a very important contributing factor in both studies, as was to be expected. In addition, Study 1 showed important effects of personality (neuroticism) and motivation (achievement). Thus, the current study suggests leads for further research on background factors that play a major role in the nomological network, in particular personality, motivation, and the experience of major life events.

Future work on the effects of appraisal bias and emotion dispositions on mood disorder should also examine relationships of this approach to the established traditions of linking locus of control, internalizing/externalizing, and attributional style with depression and anxiety (Sweeney et al., 1986; Cheng et al., 2013; Huang, 2015; Galvin et al., 2018). The major difference is that the appraisal-based emotion-focused approach does not privilege the habitual attribution of cause (external-internal) as a central determinant, but postulates a strong link between control and coping potential appraisal and an established emotion disposition as an imbalance between positive emotions on the one hand and a prevalence of sadness and worry on the other. The results of the current study show that the combination of these two predictors provides a promising estimation of potential risk for depression and anxiety. Interestingly, a similar combination of the two common temperament components of high Negative Emotionality and low Effortful Control were also strongly associated with increased psychopathology symptoms in a study by Snyder et al. (2015) with adolescents.

As mentioned with respect to the limitations of Study 1, the causal path model underlying the current investigation cannot be validated by cross-sectional studies of household panels or web surveys. Further work in this direction by using longitudinal time series approaches is urgently needed. Major efforts will be required to get appropriate longitudinal studies, which will be time- and cost-intensive, on the way. We hope that the results of the present study can help determine the priorities for the measurement of the many factors involved.

However, at this point, the evidence in the literature, confirmed by the current results, certainly encourages the urgency of developing intervention programs to minimize the adverse effects of the risk factors described here. In particular, the feasibility of health intervention activities such as increasing emotional competence and augmenting control beliefs in an effort to reduce the risk of emotional disturbances needs to be examined closely. Further work should examine in more detail which determinants are particularly valuable in reducing risk in order to better understand the underlying mechanisms and to fine-tune interventions. It would also be of interest to examine existing intervention programs for their utility in this respect. As to boosting control/coping skills, there is a plethora of programs on stress management and coping available that could be more closely evaluated in terms of the model developed here. Such projects, for example, in the framework of currently existing attempts to improve control beliefs and coping capacity (see Compas et al., 2017), should focus on promising techniques to prevent or reduce appraisal biases likely to produce dysfunctional and maladaptive emotion dispositions. Rapid development in this area is particularly urgent, given the rising incidence of depression and anxiety disorders among the young, particularly during periods of economic or health crises. This problem has become particularly salient in the enduring COVID pandemic (see Debowska et al., 2020; Kujawa et al., 2020).

## Author’s Note

The study’s design and its analysis were not preregistered. Study 1 was performed by using the data collected by the SHP, which is based at the FORS and supported by the Swiss National Science Foundation. The authors do not have any financial interest or benefit arising from the direct applications of this research.

## Data Availability Statement

The data files of the Swiss Household Panel (SHP; financed by the Swiss National Science Foundation) are available upon application to SwissUbase (https://www.swissubase.ch/en/catalogue/studies/6097/16588/overview). The Swiss Centre of Expertise in the Social Sciences (FORS) oversees the SHP. The SHP data are freely available to scientists who sign a contract agreeing to legal and ethical conditions that apply to the use of the data for research purposes (Tillmann et al., 2016). Other data files are available from the corresponding author upon reasonable request. The raw data supporting the conclusions of this article will be made available by the authors, without undue reservation.

## Ethics Statement

The studies involving human participants were reviewed and approved by the Study 1—Swiss Household Panel, Study 2—University of Bologna. The patients/participants provided their written informed consent to participate in this study.

## Author Contributions

KS has furnished the theoretical background, designed both studies, contributed to the data analysis, and wrote the first draft. V-AR has developed the control scales and performed the data analyses for the Swiss Household Survey data. PR-B and MC have designed and run the Italian survey study. All authors contributed to the article and approved the submitted version.

## Funding

This work was supported by funds granted to KS under a Swiss National Science Foundation grant (100014-122491) and a European Research Council (ERC) Advanced grant PROPEREMO (230331).

## Conflict of Interest

The authors declare that the research was conducted in the absence of any commercial or financial relationships that could be construed as a potential conflict of interest.

## Publisher’s Note

All claims expressed in this article are solely those of the authors and do not necessarily represent those of their affiliated organizations, or those of the publisher, the editors and the reviewers. Any product that may be evaluated in this article, or claim that may be made by its manufacturer, is not guaranteed or endorsed by the publisher.
