# Supplementary material for: Appraisal Bias and Emotion Dispositions Are Risk Factors for Depression and Generalized Anxiety: Empirical Evidence
Source: Front Psychol. 2022 Jul 4;13:857419. doi: 10.3389/fpsyg.2022.857419 (PMC9289678; doi:10.3389/fpsyg.2022.857419)
Supplement: Supplementary file 1 [file Presentation_1.pdf]

SOM – SUPPLEMENTAL MATERIAL ONLINE

Tables

Table S1. *Predicted Links between Dysfunctional Appraisal Biases, Frequency of Emotion Experiences, and Potential emotional disorders*

Appraisal dimension	Type of malfunction (appraisal bias)	Emotion disposition	Potential emotional disorder
Relevance detection			
Goal relevance	Inability to judge importance of events regarding goals, low intensity of motivational striving	Indifference	Apathy
Implication assessment			
Causal attribution	(i) External attribution bias<br>(ii) Internal attribution bias	(i) Anger, contempt<br>(ii) Shame, guilt	(i) Paranoia, excessive hostility<br>(ii) Shame, guilt proneness
Goal/need conduciveness	(i) Obstructiveness bias<br>(ii) Conduciveness bias	(i) Dissatisfaction<br>(ii) Satisfaction, joy	(i) Chronic dissatisfaction/ frustration<br>(ii) Euphoria
Coping potential determination			
Control			
Power	(i) Underestimation bias<br>(ii) Overestimation bias	(i) Pessimism, sadness, worry<br>(ii) Triumph	(i) Helplessness, depression, excessive anxiety<br>(ii) Mania
Normative significance evaluation			
(a) External standards	Tendency to (i) overestimate or (ii) underestimate discrepancy between own behavior and social norms	Guilt	(i) Guilt neurosis<br>(ii) Antisocial behavior
(b) Internal standards	Tendency to (i) overestimate or (ii) underestimate discrepancy between own behavior and ego ideals	Shame	(i) Shame neurosis<br>(ii) Shamelessness

Note. Adapted from Scherer and Brosch (2009), reproduced from Scherer (2021).

Table S2 Original versions of the questions concerning the central dependent variable in German, French and Italian

English	German	French	Italian
How often do you have negative feelings such as having the blues, being desperate, suffering from anxiety or depression?	Wie häufig haben Sie negative Gefühle wie Niedergeschlagenheit, Hoffnungslosigkeit, Angst oder Depressionen?	Eprouvez-vous souvent des sentiments négatifs comme le cafard, le désespoir, l'anxiété, la dépression?	Quanto spesso prova dei sentimenti negativi come la malinconia, la disperazione, l'ansia, la depressione?
How often are you full of strength, energy and optimism?	Wie häufig sind Sie voll Kraft, Energie und Optimismus?	Etes-vous souvent plein(e) de force, d'énergie et d'optimisme?	E' spesso pieno(a) di forza, di energia e di ottimismo?

Note: All rated on a 10-point scale from “never” to “always”

Table S3 Correlation matrix between central variables used in the hierarchical regressions

Pearson Correlation

	RiskMood Disorder	Sadness/ Worry	Low Coping	Gender	Age	Education	Language	Neuroticism	Achievement	Suffering
Risk_Mood Disorder	1	.489**	.484**	.108**	.035*	-.023	.106**	.350**	-.114**	.500**
Sadness/Worry	.489**	1	.394**	.149**	.097**	-.078**	.371**	.325**	-.008	.488**
Low_Coping	.484**	.394**	1	.045**	.102**	.002	.104**	.300**	-.275**	.482**
Gender	.108**	.149**	.045**	1	.034*	-.129**	-.003	.122**	-.045**	.096**
Age	.035*	.097**	.102**	.034*	1	.094**	-.018	-.041**	-.099**	.065**
Education	-.023	-.078**	.002	-.129**	.094**	1	.011	-.038*	-.029*	-.038**
Language	.106**	.371**	.104**	-.003	-.018	.011	1	.118**	.063**	.222**
Neuroticism	.350**	.325**	.300**	.122**	-.041**	-.038*	.118**	1	-.056**	.295**
Achievement motivation	-.114**	-.008	-.275**	-.045**	-.099**	-.029*	.063**	-.056**	1	-.053**
Severity of Suffering	.500**	.488**	.482**	.096**	.065**	-.038**	.222**	.295**	-.053**	1

**. Correlation is significant at the 0.01 level (2-tailed).

*. Correlation is significant at the 0.05 level (2-tailed).

Table S4 - Hierarchical regression analyses

a) Dependent Variable: Risk Mood Disorder – Depression/Anxiety (Adj. R^2 = .52)

Predictor	B	Std. Error	Beta	*t*	Sig.
(Constant)	3.706	.160		23.186	.000
MFreqSadWorry	.244	.012	.279	20.563	.000
Low_Coping	.342	.016	.266	20.818	.000
Severity of suffering	.280	.014	.270	20.207	.000
Neuroticism	.123	.011	.132	11.311	.000
Language of household	-.251	.034	-.088	-7.494	.000

b) Dependent Variable: Emotion Disposition –Sadness/Worry Disposition (Adj. R^2 = .37)

Predictor	B	Std. Error	Beta	*t*	Sig.
(Constant)	1.400	.234		5.975	.000
*Low Coping Potential	.281	.021	.192	13.463	.000
Severity of Suffering	.331	.016	.280	20.091	.000
Language in household	.900	.040	.275	22.768	.000
Neuroticism	.155	.013	.146	11.597	.000
Gender	.304	.043	.085	7.102	.000
Age	.010	.002	.078	6.518	.000
Education	-.075	.016	-.057	-4.808	.000

c) Dependent Variable: Appraisal Bias – Low Coping Potential (Adj. R^2 = .20)

Predictor	B	Std. Error	Beta	*t*	Sig.
(Constant)	-7.218	.119		-60.574	.000
Severity of Suffering	.329	.010	.406	31.633	.000
Achievement	-.190	.010	-.223	-18.101	.000
Neuroticism	.106	.009	.146	11.293	.000
Extraversion	-.084	.008	-.128	-10.234	.000
Age	.004	.001	.047	3.855	.000

Note: *entry in theoretical order, other variables entered stepwise, *s*tepping criteria: entry ($p < .001$), removal ($p > .005$).

Figures

Figure A1

Path diagram showing the results (selected predictors) of three hierarchical regression analyses: 1) Appraisal bias "Coping potential" on background variable classes a-d; 2) "Sadness/Worry" emotion disposition on appraisal bias and a-d background variable classes; and 3) Dependent variable (Depression/Anxiety risk factor for mood disorder on emotion disposition, appraisal bias, and a-d background variable classes). Paths with standardized beta weights (all ps < .001) > .10 are shown and marked with the respective beta weights. Variance explained (R^2) is bolded.

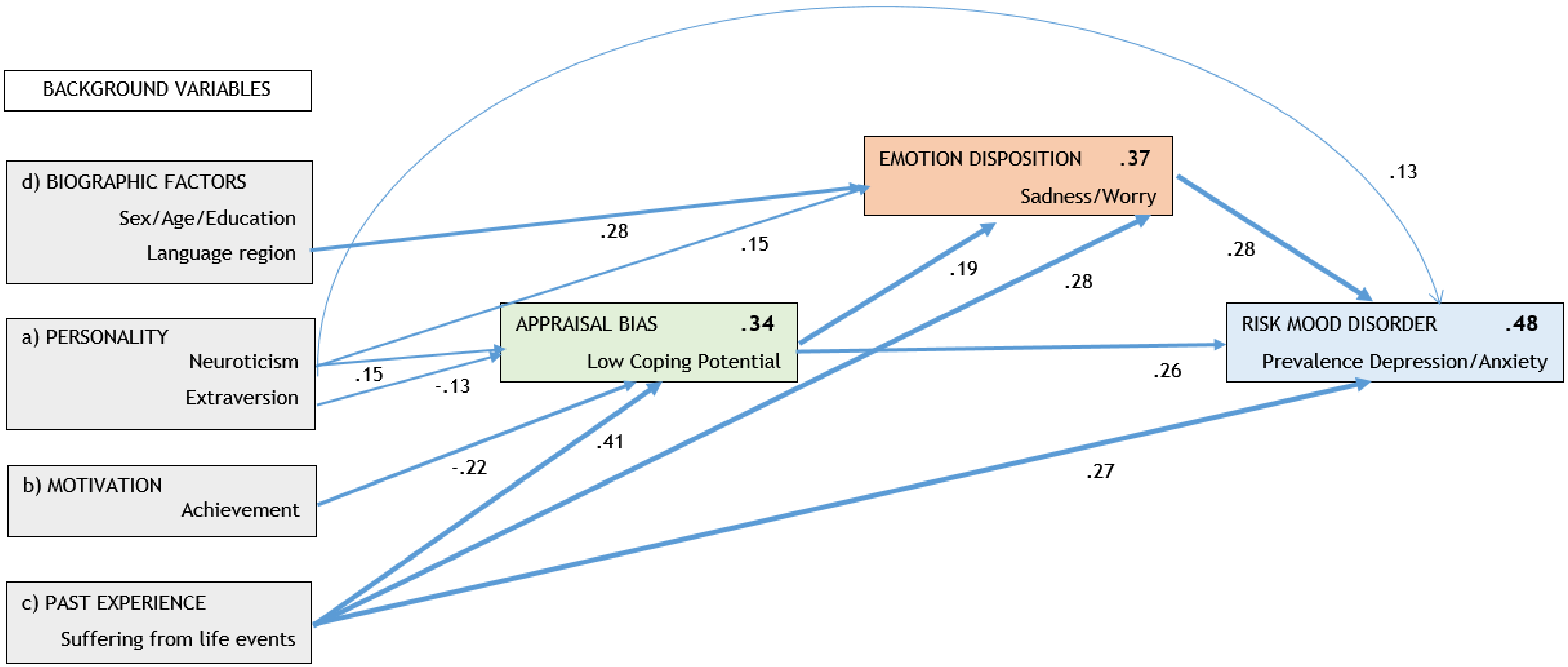

Appendix 1 – Study 1

Excerpt from an unpublished *Technical report:*

Assessing the psychometric properties of the psychological scales in the Swiss Household Panel

Development of a new Sense of Control Scale from existing items in the SHP

Analyses were performed on a subsample aged between 18 to 65 year of the 14th wave (2012) of the Swiss Household Panel (SHP) a nationwide longitudinal survey (Tillmann, et al., 2016). A total of 4858 individuals (2188 male and 2671 female) have been included in the analyses. The majority of the participants are married, have a middle level of education, and are active on the labor market. Most of the participants do not have children and the majority of individuals who have families have one to two children.

Sense of control and coping potential

Since 2012 and then every three years, a composite set of items assessed different dimensions of the sense of control and coping potential (Lachman & Weaver, 1998; Levy, Joye, Guye, & Kaufmann, 1997; Pearlin, 1981) in the SHP. These items are also used in the United States National Longitudinal Study of Health and Well-being (MIDUS) panel (Radler, 2014)

Theoretical background

Sense of control/coping potential is defined as the extent to which individuals believe that their destiny is controlled by themselves and their own decisions or by external forces over which they do not have any power. Individuals who believe more strongly that they control their own destiny are more likely to develop a feeling of self-efficacy that is beneficial for their overall subjective well-being. This is of major importance to assess the sense of control in nationwide longitudinal survey because it gauge the extent to which individual adapt to predictable or unpredictable life events or life transitions as well as the capacity individuals have to adapt to changing living conditions that are particularly measured in such survey. In addition, such measure of adaptation allows providing information in what way individuals adapt or whether they experience lack of adaptation capabilities. These lacks of resources can lead to certain forms of vulnerabilities in some subpopulation or subgroups that are important to target.

Six items measure a very general personal perception of the self. Some items measure in how far respondents believe that their destiny is controlled by themselves and their own decisions or by external forces over which they do not have any power. Individuals who believe more strongly that they control their own destiny are more likely to develop a feeling of self-efficacy.

Four questions are adapted by Levy, Joye, Guye and Kaufmann (p. 510; 1997) from Strodtbeck (1958). These items are directly related to the perception of the level of self-mastery and self-efficacy toward the environment. The last two items come from the self-esteem scale by Rosenberg (1965, adapted by Levy, Joye, Guye and Kaufmann, 1997) and reflect the appraisal of one’s own worth.

In addition, several items assessed the Personal mastery as well as the Perceived constraints. Three items come from Pearlin and Schooler (1978) and three items come from Lachman and Weaver (1998). Below the table sum up the items used in the SHP to assess one's control and coping potential.

Description of the items

An exhaustive presentation of the items, the formulation of the questions, the labels and name of the variables are displayed in tabular form below. According to Lachman and Weaver (1998), these items measure two dimensions of Sense of control and coping potential: Personal mastery and Perceived constraints.

SENSE OF CONTROL AND COPING POTENTIAL
http://www.midus.wisc.edu/midus1/documentationofscales.pdf.
Items taken from 1. Pearlin and Schooler (1978); 2. Lachman and Weaver (1998); Levy, Joye, Guye and Kaufmann adapted from Strodtbeck (1958) and from Rosenberg (1965)

PRE QUESTION

Please tell me how far you would agree with the statements I am going to read to you now, if 0 means "I strongly disagree" and 10 "I strongly agree". Please be open and honest in your responding.

VARNAME	SUB QUESTION	VARIABLE LABEL	TYPE OF VALUE
**Personal mastery**			
Lachman and Weaver, 1998			
P12C104	I can do just about anything I really set my mind to	Sense of control: Doing everything set in my mind	0: completely disagree<br>10: completely agree
P12C105	When I really want to do something, I usually find a way to succeed at it	Sense of control: Find a way to succeed	0: completely disagree<br>10: completely agree
Pearlin and Schooler, 1978			
P12C106	Whether or not I am able to get what I want is in my own hands	Sense of control: What I want is in my hands	0: completely disagree<br>10: completely agree
P12C107	What happens to me in the future mostly depends on me	Sense of control: What will happen depends on me	0: completely disagree<br>10: completely agree

Perceived constraints

Lachman and Weaver, 1998

P12C108	Other people determine most of what I can and cannot do	Sense of control: Others determine what I can do	0: completely disagree<br>10: completely agree
Pearlin and Schooler, 1978			
P12C109	I sometimes feel I am being pushed around in my life	Sense of control: Feeling of being pushed in my life	0: completely disagree<br>10: completely agree
Levy, Joye, Guye and Kaufmann adapted from Strodtbeck (1958)			
P12C70	Often it is not worth to make plans, because too much is unpredictable	incapacity to make plans because of unpredictability	0: completely disagree<br>10: completely agree
P12C71	I feel like I have little influence on the events of my life	little influence on life events	0: completely disagree<br>10: completely agree
P12C72	I am easily overcome unexpected problems	capacity to overcome unexpected problems	0: completely disagree<br>10: completely agree
P12C73	In general, I have no difficulty choosing between two possibilities	capacity to choose between two possibilities	0: completely disagree<br>10: completely agree
Rosenberg (1965)			
P12C74	Sometimes I feel useless	feeling of uselessness	0: completely disagree<br>10: completely agree
P12C75	Finally, I am rather pleased with myself	feeling of self-satisfaction	0: completely disagree<br>10: completely agree

Note: P09C72 P09C73 and P09C75 are reversed in valence.

Analyses

The model fit of this two component model was performed with the Lavaan program in the R package. As shown in Table 1, the dataset shows only marginal fit with the theoretical model. Several factors loadings shown poor correlations with the latent construct and fit indices are poor (CFI = 0.791; TLI = 0.739; RMSEA = 0.102).

Table 1. Structural Equation model based on the original theoretical model

		Estimate	Std.err	Z-value	P(>|z|)	Std.lv	Std.all
Latent variables:							
Personal mastery							
Doing everything set in my mind	P12C104	1				1.307	0.762
Find a way to succeed	P12C105	0.874	0.028	31.491	0	1.143	0.76
What I want is in my hands	P12C106	0.871	0.031	27.749	0	1.139	0.641
What will happen depends on me	P12C107	0.789	0.034	23.068	0	1.031	0.527
Perceived control							
Others determine what I can do	P12C108	1				0.934	0.427
Feeling of being pushed in my life	P12C109	1.264	0.081	15.55	0	1.18	0.538
Incapacity to make plans because of unpredictability	P12C70	0.864	0.078	11.121	0	0.807	0.308
Little influence on life events	P12C71	1.266	0.084	15.128	0	1.182	0.507
Capacity to overcome unexpected problems	P12C72	-0.819	0.065	-12.654	0	-0.764	-0.371
Capacity to choose between two possibilities	P12C73	-0.816	0.071	-11.527	0	-0.762	-0.324
Feeling of uselessness	P12C74	1.395	0.086	16.193	0	1.302	0.592

Feeling of self-satisfaction	P12C75	-1.081	0.067	-16.024	0	-1.009	-0.577

A second set of analyses was conducted based on a preliminary explanatory factor analysis and a theoretical perspective based on established concepts in the domain and the wording of the items. One item (P12C108) was removed because of the ambiguity of the formulation. This revised model postulates the following five theoretical subscales to measure sense of control and coping potential:

1. **Determination**: Doing everything set in my mind; Find a way to succeed
2. **Self-efficacy**: Capacity to overcome unexpected problem; Capacity to choose between two possibilities.
3. **Lack of agency conviction**: Incapacity to make plans because of unpredictability; Little influence on life events
4. **Power**: What I want is in my hands; What will happen depends on me.
5. **Lack of self-esteem**: Feeling of uselessness; Feeling of self-satisfaction; Feeling of being pushed in my life.

Table 2 presents the results for this new theoretical model, demonstrates good to excellent factors loadings and good model fit indices (CFI = 0.965; TLI = 0.943; RMSEA = 0.049). Based on these results, the psychometric properties of this new sense of control and coping potential scale with five subscales is considered as good.

Table 2: Structural Equation model based on the modified theoretical model

	Estimate	Std. Err.	Z-value	P(>|z|)	Std.lv	Std.all
Latent variables:						
**Determination**						
P12C104	1				1.303	0.758
P12C105	0.916	0.029	31.279	0	1.194	0.796
**Self-efficacy**						

P12C72	1				1.401	0.675
P12C73	1.006	0.072	14.002	0	1.41	0.596
**Lack of agency conviction**						
P12C70	1				1.219	0.464
P12C71	1.82	0.162	11.222	0	2.218	0.951
**Power**						
P12C106	1				1.492	0.836
P12C107	0.848	0.038	22.343	0	1.266	0.632
**Lack of self-esteem**						
P12C74	1				1.401	0.635
P12C75	-0.767	0.036	-21.511	0	-1.074	-0.611
P12C109	0.821	0.042	19.559	0	1.15	0.521

We combined the items in each subscale (mean values corrected for direction) to obtain 5 subscale variables for the control/coping construct which were used in study 1 for an exploration of the overall data structure. We computed a principal components analysis of these 5 subscale variables to obtain an indication of the underlying dimensionality. The analysis of the data in Study 1 yielded only a single factor with an Eigenvalue > 1 explaining 42.1% of the variance.

Component Matrix[a]

	Component
	1
control : determination	.797
control : self-esteem	.717
control : power	.690
control : self-efficacy	.497

control : lack of control	-.484

We then computed the mean of the subscales loading highly (> .5) on this single factor, the determination, self-esteem, and power subscales, to create a composite appraisal bias variable of *low self-perceived control/coping potential* (inverted scale). A reliability analysis yielded a Cronbach's alpha of .66 for the mean of three combined subscale variables (based on seven individual items), indicating an acceptable level of reliability.

Appendix 2 – Study 2

Given the limitation of items imposed by the feasibility of the web survey conducted for Study 2, we reduced the number of 11 items in five subscales to assess control and coping ability beliefs to 5 items.
Based on a number of criteria, independently of the subscales identified earlier, we chose the following 5 items (controlcoping) for inclusion in the study:

1 I feel like I have little influence on the events of my life
2 Often it is not worthwhile to make plans, because too much is unpredictable
3 Sometimes I feel useless
4 I feel I am sometimes being pushed around in my life
5 I easily overcome unexpected problems

A PCA of the data collected in Study 2 yields the following result (with Eigenvalue > 1 as the extraction criterion and Varimax rotation:

Rotated Component Matrix[a]

	Component	
	1	2
Controlcoping 1	.677	.446
Controlcoping 2	.789	-.221
Controlcoping 3	.484	.686
Controlcoping 4	.606	.287
Controlcoping 5	.057	-.880

Extraction Method: Principal Component Analysis.
Rotation Method: Varimax with Kaiser Normalization.
a. Rotation converged in 3 iterations.

We computed two scale values, one based on the four items positively loading on the first factor and one using five items and reversing the value of the item loading on the second factor. As the two scales correlate to r=.97, we decided to use the homogeneous four-item scale for further analyses. A reliability analysis yielded Cronbach's alpha = .66 for these four items.

Appendix 3

List of 9 Scenarios for the Emotion-Disposition-Index in Study 2

1. You have to work together with a colleague to finish an important project. He imposes his views on how to proceed and keeps criticizing your work.

2. **You have trouble getting up in the morning. Because of this, you are frequently late to arrive at the office. Today you were pretty late again. Just as you walked in, your boss passed you in the hall. Now his secretary has just telephoned to tell you that he wants to see you.**

3. You are 54 years old. Because of a company merger you have lost your position, and it has been difficult to find equivalent employment. You are given the impression that it is your age that makes people hesitate to even consider you for a position. You have just returned home from another unsuccessful interview.

4. **Your relationship with your long-term partner has been troubled lately. Your feelings toward your partner are no longer as warm as they used to be. You are wondering whether it might not be better to end the relationship.**

5. **During a party in your house, you happen to overhear two of your friends say some rather unpleasant things about you.**

6. Last week you were finally able to arrange an appointment with the head of your company to talk about a salary raise. You have just gotten home from work when you realize that you totally forgot about that meeting. It had been scheduled for this afternoon.

7. **You have been suspecting for some time that your partner might not be completely faithful to you. You now hear from an acquaintance that he has seen your partner holding hands with someone else.**

8. In a heated discussion, you made some disparaging remarks about a friend. You now have the impression that this person avoids you.

9. In the last few days, you have been spending a lot of time trying to master a difficult problem. It looked like you had finally found the solution, but it now turns out that you overlooked an important fact.

Appendix 4

Emotion Review Vol 15, number 3

SPECIAL SECTION: NORMAL AND ABNORMAL EMOTIONS—THE QUANDARY OF DIAGNOSING AFFECTIVE DISORDER

- Introduction: Special Section: Normal and Abnormal Emotions – The Quandary of Diagnosing Affective Disorder
- *Klaus Scherer and Marc Mehu*

Diagnostic classification systems – uses and problems

- Classification of Psychiatric Disorders: Challenges and Perspectives
  Norman Sartorius

- The DSM 5, and the Continuing Transformation of Normal Sadness into Depressive Disorder
  Allan V. Horwitz

Functions of sadness and anxiety – normal and abnormal

- In Between Ordinary Sadness and Clinical Depression
  Guido Bondolfi, Viridiana Mazzola, and Giampiero Arciero

- Normal and abnormal anxiety in the age of DSM-5 and ICD-11
  Dan J. Stein and Randolph Nesse

- An evolutionary approach to emotion in mental health with a focus on affiliative emotions.
  Paul Gilbert

- When and Why are Emotions Disturbed? Suggestions Based on Theory and Data from Emotion Research
  Klaus R. Scherer

Mechanisms underlying abnormal emotion processes

- An Alternative Transdiagnostic Mechanistic Approach to Affective Disorders Illustrated with Research from Clinical Psychology
  Edward Watkins
-
- Processing of Emotional Information in Major Depressive Disorder: Toward a Dimensional Understanding
  Katharina Kircanski and Ian H. Gotlib

- Emotion and the Joint Structure of Personality and Psychopathology
- *David Vachon and Robert F. Krueger*
-
- The Appraisal Bias Model of Cognitive Vulnerability to Depression
- *Marc Mehu and Klaus R. Scherer*
